# Fast and Robust Time Synchronization with Median Kalman Filtering for Mobile Ad-Hoc Networks

**DOI:** 10.3390/s21020590

**Published:** 2021-01-15

**Authors:** Young Jeon, Taehong Kim, Taejoon Kim

**Affiliations:** School of Information and Communication Engineering, Chungbuk National University, Chungju 28644, Korea; jeony9672@cbnu.ac.kr (Y.J.); taehongkim@cbnu.ac.kr (T.K.)

**Keywords:** time synchronization, ad-hoc network, fast median, Kalman filter

## Abstract

Time synchronization is an important issue in ad-hoc networks for reliable information exchange. The algorithms for time synchronization in ad-hoc networks are largely categorized into two types. One is based on a selection of a reference node, and the other is based on a consensus among neighbor nodes. These two types of methods are targeting static environments. However, synchronization errors among nodes increase sharply when nodes move or when incorrect synchronization information is exchanged due to the failure of some nodes. In this paper, we propose a synchronization technique for mobile ad-hoc networks, which considers both the mobility of nodes and the abnormal behaviors of malicious or failed nodes. Specifically, synchronization information extracted from a median of the time information of the neighbor nodes is quickly disseminated. This information effectively excludes the outliers, which adversely affect the synchronization of the networks. In addition, Kalman filtering is applied to reduce the synchronization error occurring in the transmission and reception of time information. The simulation results confirm that the proposed scheme has a fast synchronization convergence speed and low synchronization error compared to conventional algorithms.

## 1. Introduction

An ad-hoc network is adopted for various situations, such as environmental monitoring, military operation, and disaster recovery [1]. In an ad-hoc network, the nodes are equipped with computing and sensing devices operating at low power, and an accurate time synchronization is required in collecting and sharing data measured by the sensing devices [2,3]. An accurate time synchronization is a core functionality for distributed information gathering and control. For instance, in measuring the occurrence of acoustic or seismic signals over multiple probing nodes, the performance of time synchronization over the probing nodes greatly affects the accuracy of the measurement. For a high-rate time division multiple access (TDMA) system, the performance of medium access control (MAC) layer scheduling is also significantly affected by time synchronization among nodes. In addition, time synchronization plays an important role for a distributed logging system, network security, and power management system.

In a cellular communication system, mobile nodes are synchronized according to the preamble signal transmitted from a base station. Usually, a base station has enough power to send this signal, which covers the whole cell site of the base station. However, in an ad-hoc network without a base station, the available radio resources and the power for each node are scarce and limited [4,5]. Accordingly, this network requires an efficient synchronization algorithm that is robust against environmental changes along with low power consumption in the synchronization message exchanges [6].

One of the representative synchronization methods for ad-hoc networks is a reference node-based algorithm, which includes Flooding Time Synchronization Protocol (FTSP) [7] and PulseSync [8]. In this method, one of the nodes in a network is selected as a reference node, and the time of the reference node becomes a global network time. Hence, the time synchronization messages are traversed from the reference node to all the nodes in the network. The nodes receiving this synchronization message should estimate the global network time because of the time gap between transmitting and receiving a time synchronization message. Then, they update their own time information. Afterwards, the updated synchronization messages are transmitted to their neighbor nodes. All nodes in the network are synchronized by repeating this process. Various delays and errors occur in the process of receiving and transmitting the synchronization messages. In FTSP, MAC-layer timestamping is used to remove delays that occur when a synchronization message passes through communication layers [9,10,11]. However, when transmitting a message to a neighbor node, a hop delay is inevitable in every hop [12,13]. Hop delay is dependent on the distance between nodes and the message processing time. As the maximum hop count among nodes is high in a widely distributed network, hop delay remains the most important issue in the reference node-based time synchronization method [14].

Another synchronization method is a consensus-based algorithm, where each node exchanges synchronization messages with its neighbor nodes, and the synchronization message contains the time information reflecting the time information of its neighbor nodes [15]. Gradient Time Synchronization Protocol (GTSP) [16], Consensus-based Clock Synchronization (CoSyn) [17], and Random Broadcast-based Distributed consensus clock Synchronization (RBDS) [18] belong to the consensus-based synchronization algorithm. GTSP achieves time synchronization by averaging the relative rate and offset among nodes. Specifically, each node exchanges synchronization messages with its neighboring nodes in every round and stores the received time information in a table. At the end of each round, its time information is updated by averaging the rates and offsets stored in the table. The repetition of this process results in the time synchronization among all the nodes. However, if the nodes are deployed in a large area, the number of rounds required in achieving the synchronization increases sharply [19,20]. Moreover, as the number of nodes in a network increases, some nodes may malfunction due to hardware or software failure, which deteriorates the performance of time synchronization.

Naturally, a time synchronization algorithm needs to have a fast convergence in achieving consensus among nodes and to exclude malfunctioning nodes from the synchronization process. In order to achieve these goals, the median value of the time information of the nodes is adopted [21,22], because, in excluding outliers, a median—rather than an average—is a better choice.

In this paper, for mobile ad-hoc networks (MANETs) [23,24], we propose a consensus-based Median Kalman-filtering Time Synchronization (MKTS) scheme, which reduces the convergence time of synchronization by rapidly spreading the time information extracted from the median values of synchronization messages to an entire network [25]. Moreover, the proposed scheme uses a Kalman filter [26,27,28,29] in processing the synchronization messages to effectively remove the errors occurring in the synchronization. Since the median values may vary according to the location where each node is located, a Fast-median value with a reduced regional dependence is proposed. Both the mobility of nodes and the failure of nodes are considered to evaluate the performance of MKTS. The proposed algorithm has an excellent performance in mobile environments. Simulation results show that the proposed scheme has a fast convergence speed and robustness against its environmental changes. Specifically, a joining of new nodes, a removal of existing nodes, and a failure of some nodes rarely affect the performance of the proposed scheme. The target precision level of MKTS is 20 μs for both the static and mobile scenarios, and the synchronization criteria for other protocols are presented in [30,31].

The remainder of this paper is organized as follows: Section 2 describes the system model and the process of MKTS synchronization algorithms. Section 3 compares the performances of MKTS, FTSP, and GTSP in static and mobile environments with some failed nodes. Section 4 discusses the evaluation results and a future research topic, and Section 5 concludes the paper.

## 2. Proposed Time Synchronization Method

### 2.1. Clock Model

A hardware clock embedded into a node is expressed as Equation (1)

(1) $$H_{i}\left(t\right)=h_{i}\times t+o_{i},$$

where t is the actual time, $h_{i}$ is the rate of the hardware clock, and $o_{i}$ is the offset of the hardware clock. The speed of the hardware clock has an error of $\left|h_{i}-1\right|$. In manufacturing a clock counter, it is impossible to make an infinitely accurate one. An actual clock rate will be a little bit faster or slower than a perfect clock. Similarly, the sensitivity of a clock rate to ambient temperate cannot be perfectly regulated. Likewise, $h_{i}$ and $o_{i}$ are inherent characteristics of the hardware of node *i* and cannot be read or modified by node *i*. Hence, a logical clock is defined and adjusted to achieve synchronization among nodes. The logical clock $L_{i}\left(t\right)$ of node *i* is described in Equation (2).

(2) $$L_{i}\left(t\right)=l_{i}H_{i}\left(t\right)+\beta_{i}=l_{i}h_{i}t+l_{i}o_{i}+\beta_{i},$$

where $l_{i}$ is the relative logical clock rate representing the relative rate ratio of the hardware clock and the logical clock, $\beta_{i}$ is the logical offset of node *i*. Logical clock is adjusted by updating $l_{i}$ and $\beta_{i}$. In Equation (2), $x_{i}=l_{i}h_{i}$ is denoted as an absolute logical clock rate, and when all the nodes have the same absolute logical clock rate, i.e., with N nodes in a network and $x_{1}=x_{2}=\cdots =x_{N}$, the clock rate synchronization for the entire network is achieved. In the case of GTSP, the update of $x_{i}$ requires the access to the unreadable parameter $h_{i}$. Accordingly, instead of directly updating $x_{i}$, $l_{i}$ is adjusted as follows:

(3) $$l_{i}\left(t_{n+1}\right)\leftarrow \frac{\sum_{j\in N_{i}}\frac{x_{j}\left(t_{n}\right)}{h_{i}\left(t_{n}\right)}+l_{i}\left(t_{n}\right)}{\left|N_{i}\right|+1},$$

where $t_{n}$ is the time instant receiving a synchronization message from a neighbor node in the *n*th round, $N_{i}$ is a set of neighboring nodes of node *i*. In GTSP, the synchronization for the offset uses a similar method of the rate synchronization as follows:

(4) $$\beta_{i}\left(t_{k+1}\right)\leftarrow \beta_{i}\left(k_{k}\right)+\frac{\sum_{j\in N_{i}}\left(L_{j}\left(t_{k}\right)-L_{i}\left(t_{k}\right)\right)}{\left|N_{i}\right|+1}.$$

where Equations (3) and (4) are versions of the GTSP update schemes, rephrased.

### 2.2. MKTS Message and Table Structure

**Figure 1:**
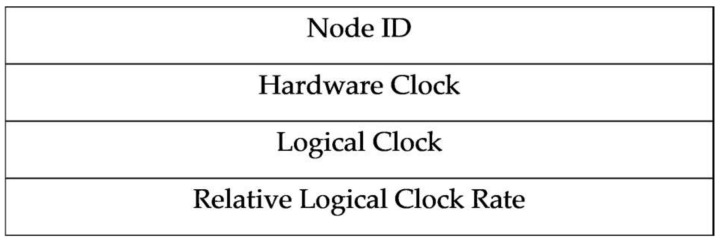
Median Kalman-filtering Time Synchronization (MKTS) packet structure.

Each node has a unique ID, and the structure of the synchronization message of MKTS is shown in Figure 1.

**Figure 2:**
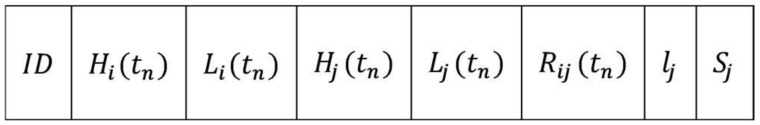
MKTS message table structure.

The table structure for node *i* to store the information received from node *j* is shown in Figure 2. Receiving a new message, a new row is added.

The ID of node *j* is stored at the first field. $H_{j}\left(t_{n}\right)$ is the hardware clock of node *j* received in the *n*th round, and $H_{i}\left(t_{n}\right)$ is the hardware clock of node *i* measured at the instant of receiving $H_{j}\left(t_{n}\right)$ in the *n*th round. $L_{j}\left(t_{n}\right)$ is the logical clock received from node *j*. $L_{i}\left(t_{n}\right)$ is the logical clock of node *i* measured at the instant of receiving $L_{j}\left(t_{n}\right)$ in the *n*th round. $R_{ij}\left(t_{n}\right)$ is the relative hardware clock rate in the *n*th round, which is the ratio of hardware clock rate of node *j* to the hardware clock rate of node *i*. $l_{j}$ is the relative logical clock rate of node *j*. $S_{j}$ is the number of messages received from node *j*.

### 2.3. Relative Hardware Clock Rate

As shown in Equation (3), $\frac{x_{j}\left(t_{n}\right)}{h_{i}\left(t_{n}\right)}$ is required in synchronizing the relative logical clock rate $l_{i}$. Without access to the unreadable $h_{i}\left(t_{n}\right)$, $\frac{x_{j}\left(t_{n}\right)}{h_{i}\left(t_{n}\right)}$ can be obtained as follows [32]:

(5) $$\frac{x_{j}\left(t_{n}\right)}{h_{i}\left(t_{n}\right)}=\frac{\frac{L_{j}\left(t_{n}\right)-L_{j}\left(t_{n-1}\right)}{t_{n}-t_{n-1}}}{\frac{H_{i}\left(t_{n}\right)-H_{i}\left(t_{n-1}\right)}{t_{n}-t_{n-1}}}=\frac{L_{j}\left(t_{n}\right)-L_{j}\left(t_{n-1}\right)}{H_{i}\left(t_{n}\right)-H_{i}\left(t_{n-1}\right)}=\frac{\Delta L_{j}}{\Delta H_{i}}=\frac{\Delta H_{j}l_{j}}{\Delta H_{i}}=\frac{H_{j}\left(t_{n}\right)-H_{j}\left(t_{n-1}\right)}{H_{i}\left(t_{n}\right)-H_{i}\left(t_{n-1}\right)}\times l_{j}.$$

$R_{ij}\left(t_{n}\right)$ is expressed as Equation (6).

(6) $$R_{ij}\left(t_{n}\right)=\frac{h_{j}\left(t_{n}\right)}{h_{i}\left(t_{n}\right)}=\;\frac{\frac{H_{j}\left(t_{n}\right)-H_{j}\left(t_{n-1}\right)}{t_{n}-t_{n-1}}}{\frac{H_{i}\left(t_{n}\right)-H_{i}\left(t_{n-1}\right)}{t_{n}-t_{n-1}}}=\frac{H_{j}\left(t_{n}\right)-H_{j}\left(t_{n-1}\right)}{H_{i}\left(t_{n}\right)-H_{i}\left(t_{n-1}\right)}.$$

Hence, $\frac{x_{j}\left(t_{n}\right)}{h_{i}\left(t_{n}\right)}=R_{ij}\left(t_{n}\right)l_{j}$ is satisfied. In order to increase the accuracy of $R_{ij}\left(t_{n}\right)$, the errors caused by delay are reduced by adopting an integral filter [33], which takes the weighted moving average for the continual input of $R_{ij}\left(t_{n}\right)$. Since the weighted current $R_{ij}\left(t_{n}\right)$ is added to the previous average, the error can be filtered out. The filtered version $R_{ij}^{\prime }\left(t_{n}\right)$ can be obtained as Equation (7).

(7) $$R_{ij}^{\prime }\left(t_{n}\right)=\frac{\min\left[S_{j},5\right]-2}{\min\left[S_{j},5\right]-1}\times R_{ij}^{\prime }\left(t_{n-1}\right)+\frac{1}{\min\left[S_{j},5\right]-1}\times R_{ij}\left(t_{n}\right),\;S_{j}\geq 2.$$

If the maximum $S_{j}$ is very high, the averaging is as good as being taken over by very many previous $R_{ij}\left(t_{n}\right)$s, and the impact of the current $R_{ij}\left(t_{n}\right)$ will be marginal. Accordingly, a high $S_{j}$ results in a good performance in eliminating error; however, too high $S_{j}$ may result in the deterioration of catching the actual changes of $R_{ij}\left(t_{n}\right)$ like clock rate changes from ambient temperature change, which should be reflected as not being filtered out. In selecting the maximum $S_{j}$, FTSP, which adopts least square method using 4–8 previously received messages is referred. Since the proposed method is focused on mobile scenarios, relatively small $S_{j}=5$ is selected as a maximum value.

### 2.4. Update Rule

When a node receives synchronization messages from its neighbor nodes, it may update its time information to an average or a consensus value of the received time information. Alternatively, it can be synchronized to the time of a specific node [34]. For instance, if node *i* determines to follow the clock of node *j*, the absolute logical clock rate and logical clock of node *i* must be modified to the values of node *j*. Accordingly, $x_{i}\left(t_{n}\right)\leftarrow x_{j}\left(t_{n}\right)$ and $L_{i}\left(t_{n}\right)\leftarrow L_{j}\left(t_{n}\right)$ should be achieved by adjusting $l_{i}$ and $\beta_{i}$ as shown in Equations (8) and (9).

(8) $$l_{i}\leftarrow R_{ij}\left(t_{n}\right)\times l_{j}=\frac{h_{j}}{h_{i}}\times l_{j}=\frac{x_{j}}{h_{i}},$$

(9) $$\beta_{i}\leftarrow L_{j}\left(t_{n}\right)-l_{i}\times H_{i}\left(t_{n}\right).$$

In MKTS, each node updates its clock information by selecting a specific target node and changing its time information following the selected one. The criterion of selecting the target node is an integral part of the proposed scheme, and the detailed selection process is described in what follows. Basically, the selected target node has a median logical clock from among its neighboring nodes. This method plays an important role in excluding outliers and expediates the convergence of the synchronization process. Kalman filtering follows this median-based approach to reduce the errors caused by noise and delay. Using both the median-based approach and Kalman filtering, a robust time synchronization is achieved against the mobility of the nodes in a MANET.

**Figure 3:**
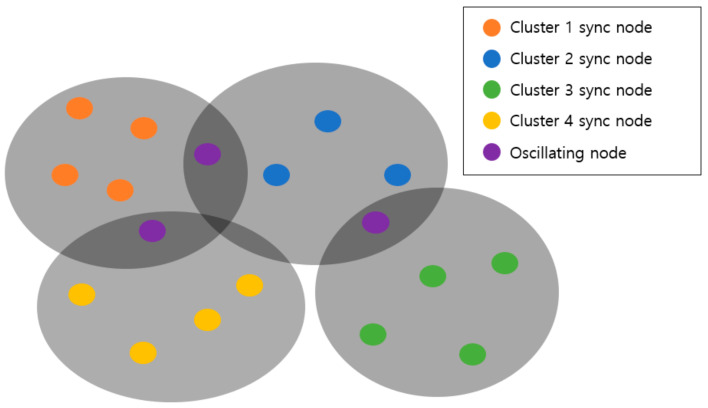
Exemplified network with four clusters and oscillating nodes.

However, directly adopting a medium of logical clocks from among neighbor nodes can cause a problem. Since each node selects a medium node from among its own 1-hop neighboring nodes, if the nodes of a MANET are distributed over a large area, the logical clocks of the selected mediums will be different depending on the geographic location of each node. Accordingly, oscillations may occur in exchanging the selected medium logical clocks at each synchronization round. For instance, as shown in Figure 3, there are four clusters represented as gray areas. The clusters with orange nodes and blue nodes have a single intersection node colored purple. This purple node can be synchronized to the cluster with orange nodes at one round, and it can be synchronized to other cluster with blue nodes at some other round. This causes oscillation to the purple node. As many rounds pass, this oscillation will be diminished; however, if the nodes are deployed over a wide area, the convergence will take a much longer time. These oscillations hinder the synchronization and slow down the convergence. Therefore, instead of adopting the logical clock of the selected median node, Fast-median (F-Median) is proposed. F-Median selects the fastest logical clock within a certain range, where the range is centered at the logical clock of the medium node and has a predefined span. F-Median can reduce the oscillation while excluding outliers.

**Figure 4:**
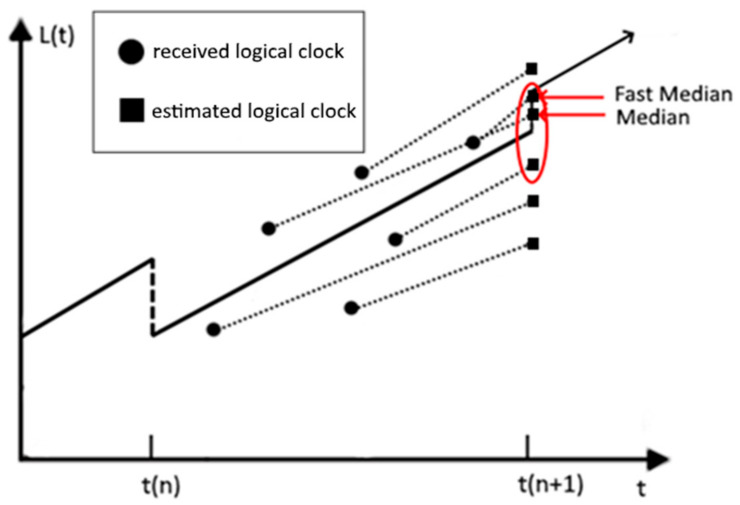
Median value in synchronization round.

Figure 4 depicts F-Median in the *n*th synchronization round, where $t\left(n\right)$ is the start time of the *n*th synchronization round, black circles are the received logical clocks, and the black squares are the estimated logical clocks at the synchronization round boundary.

In order to select a node with a median logical clock from among neighboring nodes, the exact logical clocks at the synchronization round boundary $t\left(n+1\right)$ should be estimated. When node *i* receives a message from node *j* at $t_{n}$ belonging to the *n*th round, i.e., $t_{n}\in \left[t\left(n\right),\;t\left(n+1\right)\right)$, the logical clock of node *j* at $t\left(n+1\right)$ can be estimated as follows:

(10) $$L_{j}\left(t\left(n+1\right)\right)=L_{j}\left(t_{n}\right)+\left(H_{i}\left(t\left(n+1\right)\right)-H_{i}\left(t_{n}\right)\right)\times R_{ij}\left(t_{n}\right)\cdot l_{j}.$$

After the estimations, the neighboring nodes are sorted according to the estimated logical clocks. Subsequently, a medium node is selected from the sorted neighboring nodes. The process of selecting the median node can be expressed as follows:

(11) $$L_{i}^{Med}\left(t\left(n+1\right)\right)=Median_{v\in N_{i}}(L_{v}\left(t\left(n+1\right)\right),$$

where $L_{i}^{Med}\left(t\left(n+1\right)\right)$ is the median value selected by node *i* at $t\left(n+1\right)$, and $Median_{v\in N_{i}}\left(\cdot \right)$ is a function that returns the median value from the 1-hop neighbor node set $N_{i}$ of node *i*. Then, a range centered at the selected logical clock with the predefined span is determined, and the node with the fastest logical clock within that range is finally selected. The detailed F-Median process is described in Algorithm 1.
**Algorithm 1.** Fast Median.**Input:** THRESHOLD: upper bound of F-Median range**Output:**1: **for each**
$j\in N_{i}$2: $L_{j}\left(t\left(n+1\right)\right)\leftarrow L_{j}\left(t_{n}\right)+\left(H_{i}\left(t\left(n+1\right)\right)-H_{i}\left(t_{n}\right)\right)\times R_{ij}\left(t_{n}\right)\times l_{j}$3: **end**4: ${\mathrm{Sort}}_{v\in N_{i}}\left(L_{v}\left(t\left(n+1\right)\right)\right)$5: $M\leftarrow \arg\;Median_{v\in N_{i}}(L_{v}\left(t\left(n+1\right)\right)$6: $\mathrm{for}\;\mathrm{each}\;j\in N_{i}$7:   $e\leftarrow L_{M}\left(t\left(k+1\right)\right)-L_{j}\left(t\left(k+1\right)\right)$8:   **if**
e<THRESHOLD9:   Fastest_Median_Value←j10:    break11:   **end**12: **end**

Kalman filtering follows the F-Median process to remove the errors due to topology change or randomness in transmission and reception. If node *i* selects node *j* as a F-Median node, the input to the Kalman filter can be expressed as Equations (12) and (13) [35].

(12) $$T_{n}=L_{i}\left(t_{n}\right)-L_{j}\left(t_{n}\right),$$

(13) $$\begin{array}{l} D_{n}=\frac{x_{j}\left(t_{n}\right)-x_{i}\left(t_{n}\right)}{x_{i}\left(t_{n}\right)}=\frac{L_{j}\left(t_{n}\right)-L_{j}\left(t_{n-1}\right)}{L_{i}\left(t_{n}\right)-L_{i}\left(t_{n-1}\right)}-1\\ =\frac{l_{j}\times \left(H_{j}\left(t_{n}\right)-H_{j}\left(t_{n-1}\right)\right)\times t}{l_{i}\times \left(H_{i}\left(t_{n}\right)-H_{i}\left(t_{n-1}\right)\right)\times t}-1=\frac{R_{ij}l_{j}}{l_{i}}-1, \end{array}$$

where $T_{n}$ is the logical clock difference between node *i* and node *j* in the *n*th round. $D_{n}$ is the ratio of the absolute logical clock rate difference between node *i* and node *j* to the absolute logical clock rate of node *i* in the *n*th round. $x_{n}$ is a column vector having $T_{n}$ and $D_{n}$ as elements as shown in (14).

(14) $$x_{n}=\left[\begin{array}{c} T_{n}\\ D_{n} \end{array}\right].$$

where $x_{n}$ is the observed values in the *n*th round in a real environment, and a model for the environment needs to be designed. The parameters for the environment adopted in MKTS are shown in Equations (15) and (16).

(15) $$\Delta H_{i}=H_{i}\left(t\left(n+1\right)\right)-H_{i}\left(t_{n}\right),$$

(16) $$A_{n}=\left[\begin{array}{cc} 1 & \Delta H_{i}\\ 0 & 1 \end{array}\right],$$

where $A_{n}$ is the environment model in the *n*th round. The initial input vector is $x_{0}$, and the initial covariance of the input data is arbitrarily set to $P_{0}$. From these initial values and Equations (17) and (18), $x_{p}$ and $P_{p}$ are obtained as follows:

(17) $$x_{p}=A_{n}x_{n},$$

(18) $$P_{p}=A_{n}P_{n}A_{n}^{T}+B_{n}QB_{n}^{T},$$

where $x_{p}$ is a predicted input data, and $P_{p}$ is a predicted covariance of the input data. In addition, $x_{n}$ is the actual input data of the *n*th round, and $P_{n}$ is the covariance of the actual input data. $B_{n}$ is the transformation matrix of Q, where Q is the noise matrix generated in the process of predicting the covariance shown as follows:

(19) $$Q=\left[\begin{array}{cc} \delta^{2} & 0\\ 0 & \varphi^{2} \end{array}\right],$$

where δ and φ are Gaussian noises. In order to deal with nonlinearity in the estimation, instead of Equation (18), (20) can be used [36].

(20) $$P_{p}=A_{n}P_{n}A_{n}^{T}+\left[\begin{array}{cc} 1 & \frac{\Delta H_{i}}{2}\\ 0 & 1 \end{array}\right]Q\left[\begin{array}{cc} 1 & \frac{\Delta H_{i}}{2}\\ 0 & 1 \end{array}\right]\Delta H_{i},$$

where $\Delta H_{i}$ is obtained from Equation (15). Moreover, in Equation (20), the error occurring in the process of predicting the covariance of a nonlinearly operating model is calculated using the Riccati equation. $P_{p}$ is used to calculate the Kalman gain using the relationship between the actual and predicted data as follows:

(21) $$K_{n}=P_{p}U_{n}^{T}{\left(U_{n}P_{p}U_{n}^{T}+R\right)}^{-1},$$

(22) $$U_{n}=\left[\begin{array}{cc} 1 & \Delta H_{i} \end{array}\right],$$

where $K_{n}$ is the Kalman gain in the *n*th round, R is the Gaussian noise of the observed data, and $U_{n}$ is a transformation matrix of measured values. The Kalman gain is used to adjust the actual data and the predicted data, $y_{n}$ and $y_{p}$, respectively, which are given by Equations (23) and (24):

(23) $$y_{n}=U_{n}x_{p}+u,$$

(24) $$y_{p}=U_{n}x_{p},$$

where u is noise generated in the process of measuring data. $x_{n}$ and $P_{n}$ are updated following Equations (25) and (26).

(25) $$x_{n+1}=x_{p}+K_{n}\left(y_{n}-y_{p}\right)=\left[\begin{array}{c} T_{n+1}\\ D_{n+1} \end{array}\right],$$

(26) $$P_{n+1}=\left(I-K_{n}U_{n}\right)P_{p}.$$

After updating $x_{n+1}$ and $P_{n+1}$, node *i* updates its own logical clock and relative logical clock rate as follows:

(27) $$L_{i}\left(t\left(n+1\right)\right)=L_{i}\left(t\left(n+1\right)\right)-T_{n+1},$$

(28) $$l_{i}=l_{i}\times \left(D_{n+1}+1\right).$$

**Figure 5:**
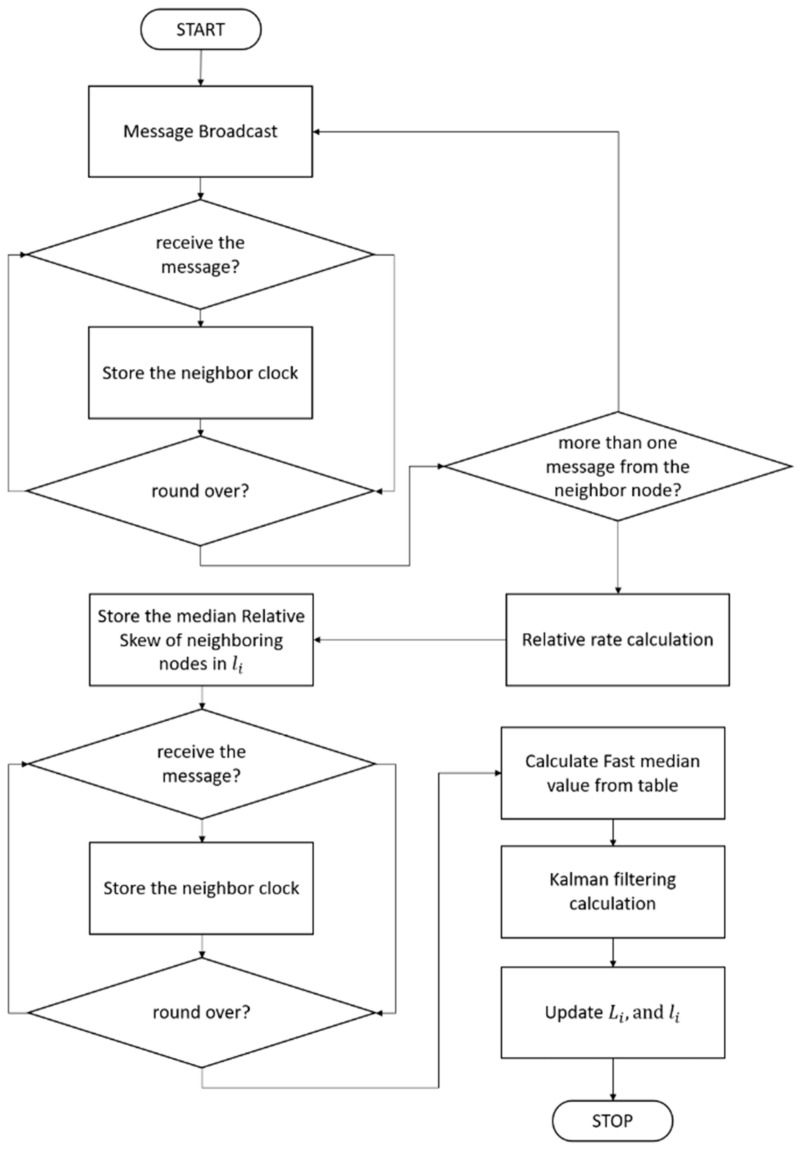
Flowchart of the whole process of MKTS.

The synchronization process is repeated every round to optimize the predicted data and covariance and to remove errors caused by noise and delay. In practical protocols, the synchronization round has the same period with beacon transmission period, and some portion of the super frame is allocated to time synchronization message exchanges. A flowchart showing the whole synchronization process is depicted in Figure 5.

## 3. Performance Evaluation

**Figure 6:**
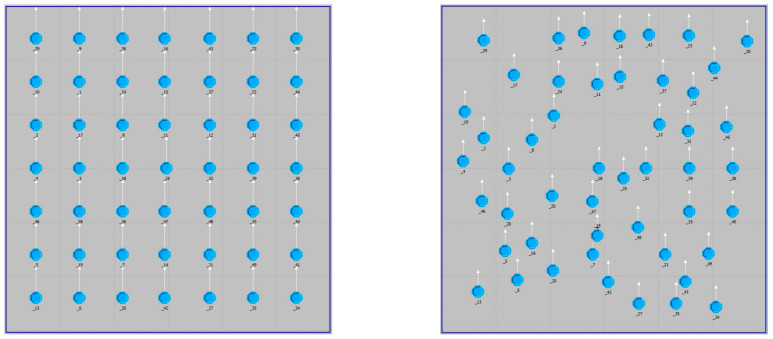
Mesh and random topologies for node placement.

The performance of MKTS is verified by comparing MKTS with other conventional algorithms of FTSP and GTSP, under the environment considering mobile nodes and node failures. The specific evaluation plan is to compare MKTS with FTSP and GTSP in static and mobile environments, to compare MKTS with FTSP and GTSP by varying the speed of nodes and the size of the network area, and finally, to evaluate MKTS with an increasing number of malfunctioning nodes. In this comparative evaluation, FTSP and GTSP are not the state-of-the-art techniques, however, they are still the representative techniques for ad-hoc network time synchronization. Moreover, the proposed scheme focuses on the mobility of nodes, which is not supported by most of the time synchronization techniques. In addition, the most consensus-based algorithms follow the basic philosophy of GTSP, i.e., each adjusts its own time information according to the average information of its neighbor nodes. Accordingly, the contribution of the proposed scheme can be evaluated by comparing with FTSP and GTSP. For the performance analysis, the network simulator OPNET [37] is used, which is an established commercial network simulator readily supporting practical protocols like IEEE 802.11 and IEEE 802.15.4. In measuring the performance of the proposed algorithm, Maximum Network Error $A_{e}$ and Maximum Neighbor Error $N_{e}$ are used, where $A_{e}$ is the largest logical clock difference among nodes, and $N_{e}$ is the largest logical clock difference between neighboring nodes in an entire network. $A_{e}$ and $N_{e}$ are expressed as follows:

(29) $$A_{e}=max_{v,w\in N}\left\{\left|L_{v}\left(t\right)-L_{w}\left(t\right)\right|\right\},$$

(30) $$N_{e}=\max_{v,w\in N_{v}}\left\{\left|L_{v}\left(t\right)-L_{w}\left(t\right)\right|\right\},$$

where N is the set of all nodes in a network, and $N_{v}$ is the set of node *v*’s 1-hop neighbor nodes. To compare the performance, the placement of the nodes is depicted in Figure 6. The parameters of the simulations are summarized in Table 1.

### 3.1. Performance Comparison with Conventional Methods

**Figure 7:**
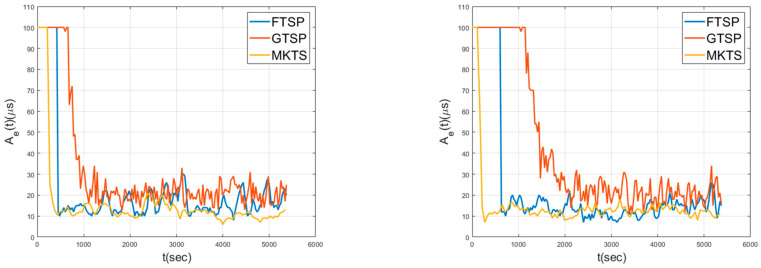
Network error in static environment for mesh (left) and random (right) topologies.

Figure 7 shows $A_{e}$ over time while the nodes are static for a mesh (left) topology and a random (right) topology. The criterion determining the achievement of the network synchronization is set as 20 μs in $A_{e}$. As shown in this figure, FTSP has an advantage in the synchronization speed because this scheme has the global reference clock to quickly disseminate over the network. On the other hand, in GTSP, since it takes relatively many rounds to calculate the average of the clocks of neighboring nodes, the convergence speed is low. Even though MKTS adopts a distributed approach, its convergence speed is faster than FTSP.

For the mesh topology, the convergence times, i.e., the times taken in achieving $A_{e}\leq 20\;\mu s$, are in the increasing order of MKTS, FTSP, and GTSP. In addition, the average $A_{e}$s for these schemes are 12.13 μs, 15.11 μs, and 16.26 μs, respectively. A similar performance is maintained for the random topology. Note that MKTS achieves the best performance both in the convergence time and $A_{e}$ under the static environment.

**Figure 8:**
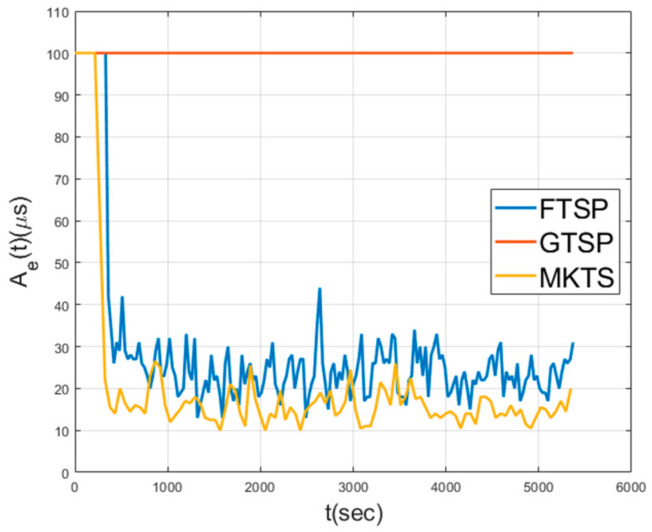
Network error in mobile environment.

Figure 8 shows $A_{e}$s in a mobile environment, where the nodes move at a speed of 5 m/s in random directions. As shown in this figure, GTSP fails in achieving the synchronization, because the members of a set of neighboring nodes change too quickly for the averaged clock value to converge. Since FTSP synchronizes with the clock of the reference node, even when the network topology is changed, it can achieve synchronization. However, note that the average $A_{e}$ is 28.7 μs, indicating that the performance decreases by 76% compared to the performance under static environment shown in Figure 7. The average $A_{e}$ of MKTS is 15.73 μs, which is much better than FTSP. Moreover, the performance decrement of MKTS compared with the static environment is 23%; therefore, it is clearly better than FTSP. In general, under a mobile environment, the decrease in the synchronization performance is inevitable because neighboring nodes may be changed in every round. However, MKTS minimizes this performance degradation by effectively excluding outliers using F-Median and by reducing clock estimation error using Kalman filtering.

### 3.2. Synchronization Performance with Varying Area Size and Node Speed

**Figure 9:**
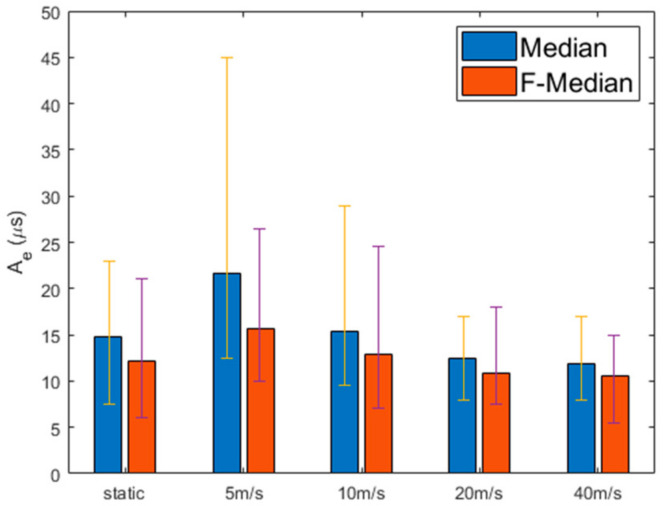
Network error with median and F-median in mobile environment.

Figure 9 compares F-Median with the median-based approach by varying the speed of the nodes, where the median approach selects a median node from among the neighboring nodes without considering the Fast-logical clocks. Both the F-Median and the simple median use Kalman filtering. This figure shows that, regardless of the speed of the nodes, F-Median has a better performance than the median-based method. In the static case, F-Median and the median-based method have the similar $A_{e}$ deviations (orange line and violet line, respectively); however, F-Median has 18% better $A_{e}$ than the median-based method. In particular, when the node speed is 5 m/s, F-Median has 49.3% lower deviation and 27.4% lower $A_{e}$ than the median-based approach.

**Figure 10:**
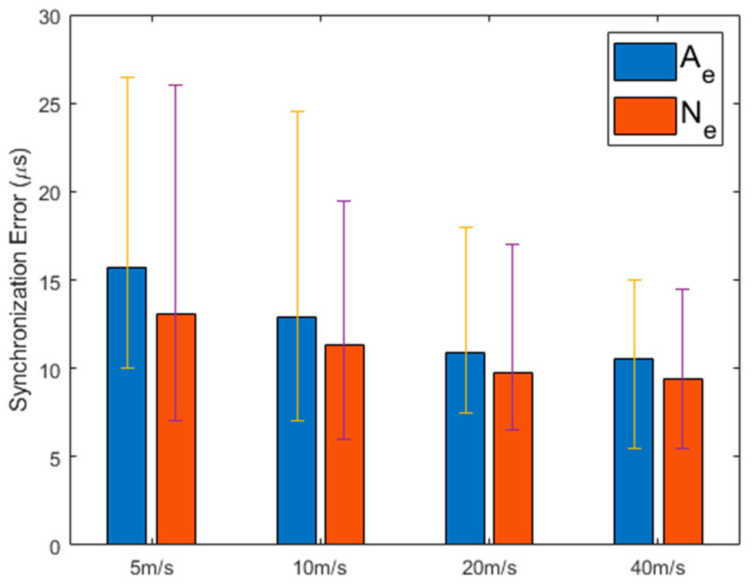
Synchronization error with varying node speed in mobile environment.

Figure 10 shows $A_{e}$ and $N_{e}$ of MKTS in a mobile environment. This figure shows that the synchronization performance is maintained even when the speed of the nodes increases. If the size of the area, where the nodes are deployed, is not large, the increasing the speed of the nodes can be beneficial in achieving the synchronization as shown in this figure. However, if the area is very large or has no boundary, as the speed of the nodes increases, the synchronization performance decreases. For instance, in an environment in which there is no boundary of limiting the movement of nodes, the higher the speed of random walking nodes, the more prone the radio link among the nodes is to be broken, resulting in a decrease in the synchronization performance.

**Figure 11:**
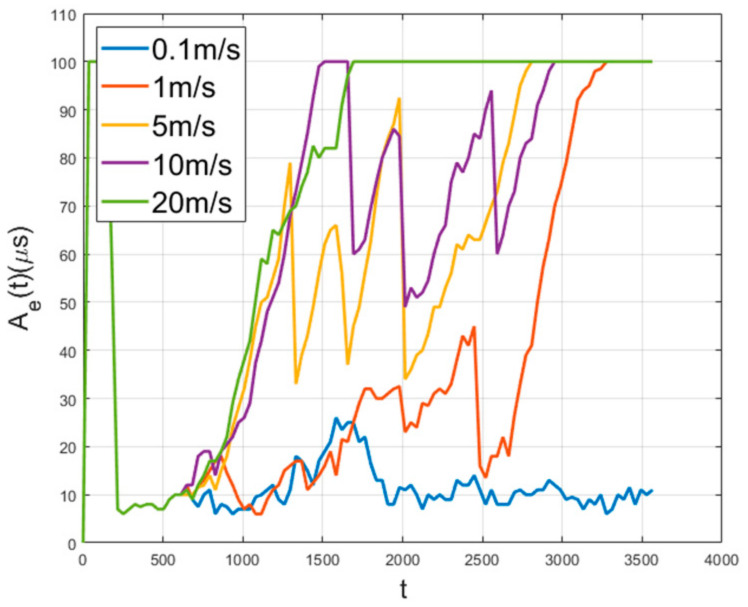
Network error with no boundary.

**Figure 12:**
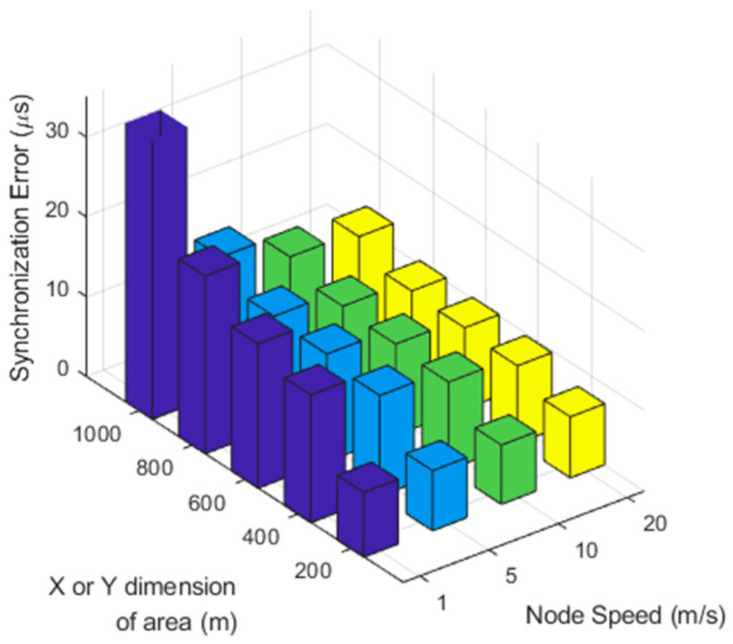
Network error with varying area size and node speed.

$A_{e}$ and $N_{e}$ for random walking without boundary is depicted in Figure 11. In this figure, the nodes are static until 600 s achieving time synchronization, after 600 s, the random walk starts. Figure 11 shows that the slower the speed, the longer the synchronization is maintained. In addition, if the speed of the nodes is high, the nodes within the synchronization boundary quickly leave it, and the synchronization performance decreases rapidly. Figure 12 shows the performance according to the various area size and the node speed. It is inversely proportional to area size and proportional to speed. As the range of the nodes’ movement gets smaller, the amount of the exchanged time synchronization messages is maintained at a high level. If the nodes are deployed over a bounded area, as the speed of the nodes increases, the probability of a node getting out of the synchronization group increases.

**Figure 13:**
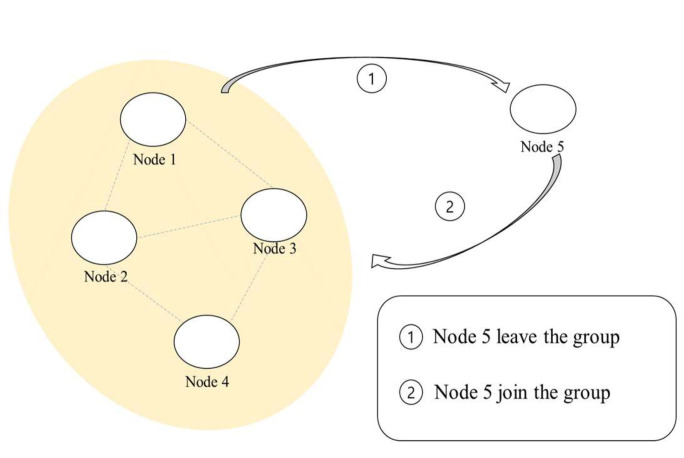
Network boundary.

However, as shown in Figure 13, if the boundary is small and the speed of the nodes high, the probability of a node entering the synchronization group also increases. On the other hand, if the speed is low, the probability of a node getting out of the synchronization group is decreased, but the probability of a node, which left the synchronization group, re-entering the synchronization group decreases, resulting in the decrease in the synchronization performance of the entire network.

### 3.3. Performance Analysis with Malfunctioning Nodes

As the number of nodes increases, the number of malfunctioning nodes may increase. If a node exhibits abnormal behavior due to hardware or software failure, the network synchronization performance is greatly reduced. MKTS improves the synchronization performance by excluding malfunctioning nodes from the synchronization process.

**Figure 14:**
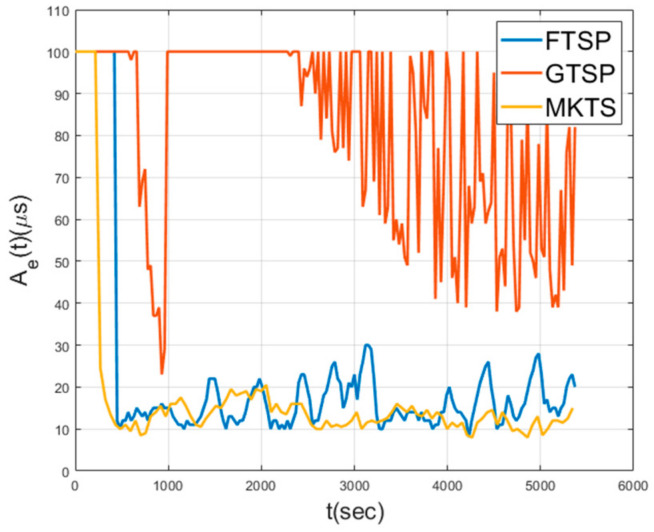
Network error with a single malfunctioning node.

**Figure 15:**
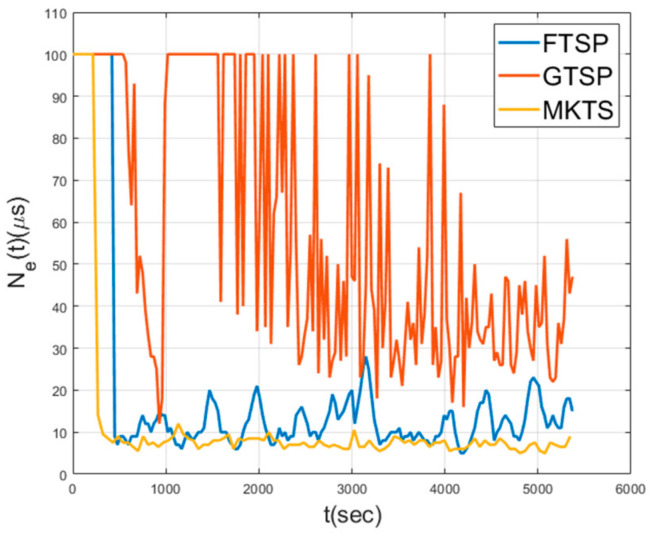
Neighbor error with a single malfunctioning node.

Figure 14 and Figure 15 show $A_{e}$ and $N_{e}$ when a single node exhibits abnormal behavior. In this simulation, a single node starts to malfunction and to send corrupted time synchronization messages at 900 s. In these figures, the synchronization performance of GTSP deteriorates and fails to achieve time synchronization. FTSP converges but shows the increased fluctuation during the simulation time. On the other hand, MKTS has very a small fluctuation and successfully maintains the synchronization, even when a malfunctioning node exists.

**Figure 16:**
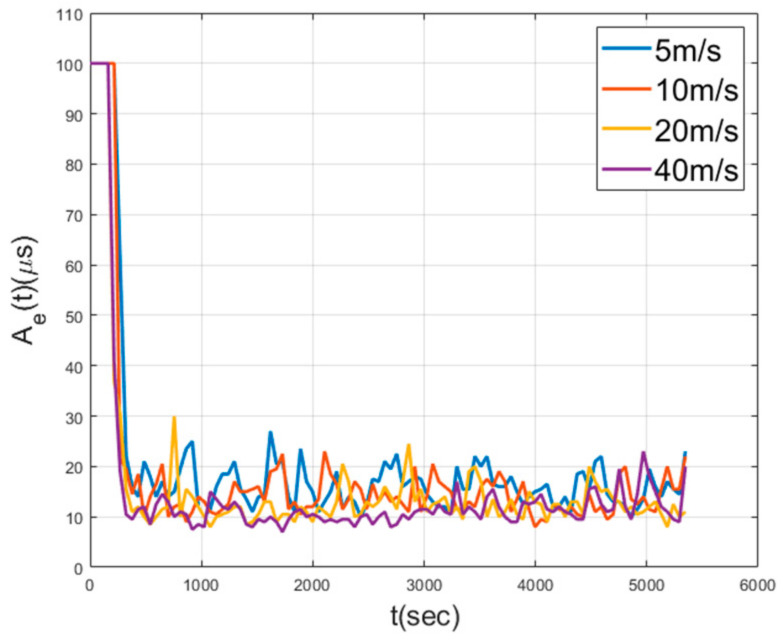
Mobile network error with 2 malfunction nodes.

**Figure 17:**
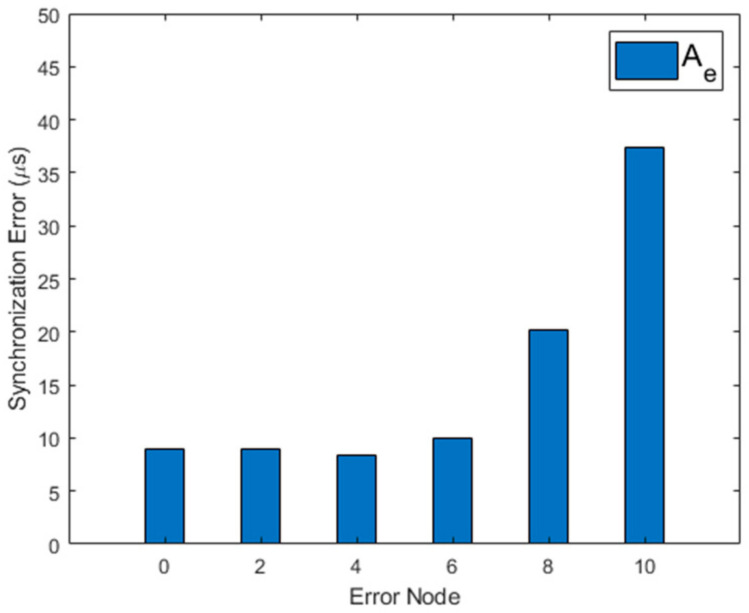
Network error with increasing malfunction nodes.

Figure 16 shows the performance of MKTS with varying speed of nodes while two malfunctioning nodes exist. In this simulation, one node fails at 600 s and the other at 1500 s, respectively. As shown in this figure, MKTS successfully achieves time synchronization by excluding these two malfunctioning nodes from the synchronization process, and the speed of the nodes hardly affects the synchronization performance. Figure 17 shows the performance of MKTS with the increasing number of malfunctioning nodes. Performance decrement is very small up to six malfunctioning nodes, i.e., 12.2% of the total nodes are malfunctioning nodes.

However, when more than six nodes are malfunctioning, the performance starts to decrease. Even if malfunctioning nodes exist, MKTS recovers the synchronization performance within a short period of time. Moreover, under the harsh environment, in which 12.2% of the nodes are transmitting corrupted time information, MKTS maintains a good time synchronization.

## 4. Discussion

The results of the performance evaluation clearly show the advantage of MKTS. Compared with conventional time synchronization protocols for both the static and mobile scenarios, the excellent performance is confirmed. In particular, the performance gap is remarkable under the mobile scenarios because the proposed scheme maintains time synchronization under the harsh condition that nodes move with high speed. Moreover, fast convergence times are achieved by efficiently disseminating Fast-median values. Another notable strength of MKTS is its robustness and resilience against environmental changes. Even when 12.2% of malfunctioning nodes transmit corrupted time synchronization messages, MKTS quickly recovers time synchronization. These results support the working hypotheses that Fast-median efficiently excludes outliers and Kalman filtering enhances the synchronization.

A drawback of the proposed scheme is that MKTS has a difficulty in finding the optimal Fast-median when there are a small number of nodes in a network. The purpose of adopting median values is to exclude outliers from samples; however, when the number of samples is small, the probability that outliers fall into the range, which is centered at a median, increases. This decreases synchronization performance of MKTS; however, note that other time synchronization scheme can also suffer from this problem if a small number of nodes are scattered over a wide area.

For a future research topic extending the proposed scheme, a time-synchronization protocol with an aerial relay node can be considered. The proposed scheme is targeting for a MANET. When a group of nodes are connected to a base station via an aerial relay node, the time synchronization will be an interesting and challenging issue extending the proposed scheme. In this case, the protocol needs to optimize the operation of the fast-moving aerial relay node, and time synchronization should take this feature into account.

## 5. Conclusions

In this paper, we propose a time synchronization algorithm for mobile environments, which removes outliers using the F-Median of synchronization messages and eliminates synchronization errors using Kalman filtering. In the case of conventional FTSP, the convergence speed of synchronization is fast, but performance decreases as the number of hops increases. In GTSP, the effect of a large hop count is small, but the convergence speed is low. In addition, when nodes in a network act abnormally due to a hardware or software failure, the network synchronization performance is greatly reduced. MKTS shows the fast convergence speed and an accurate synchronization performance. In addition, even if some nodes behave abnormally in a network, these nodes are effectively excluded from the synchronization process to maintain synchronization performance.

## Figures and Tables

**Table 1:** System parameters.

Parameter	Value
Number of Nodes	49
X Dimension	600 m
Y Dimension	600 m
Communication Range	110 m
Topology	Grid/Random
Mobility	Random Direction
Beacon Interval	30 s
Hardware Clock Drift	−30~+30 (μs)
Hardware Clock Drift Variation	−5~+5 (μs)

## Data Availability

Data is contained within the article.
